# *Bartonella vinsonii *subsp. *berkhoffii *and *Bartonella henselae *bacteremia in a father and daughter with neurological disease

**DOI:** 10.1186/1756-3305-3-29

**Published:** 2010-04-08

**Authors:** Edward B Breitschwerdt, Ricardo G Maggi, Paul M Lantos, Christopher W Woods, Barbara C Hegarty, Julie M Bradley

**Affiliations:** 1 Intracellular Pathogens Research Laboratory, Center for Comparative Medicine and Translational Research, College of Veterinary Medicine, North Carolina State University, 4700 Hillsborough St., Raleigh, NC, USA; 2 Duke University Medical Center, 2301 Erwin Rd, Durham, NC, USA

## Abstract

**Background:**

*Bartonella vinsonii *subsp. *berkhoffii *is an important, emerging, intravascular bacterial pathogen that has been recently isolated from immunocompetent patients with endocarditis, arthritis, neurological disease and vasoproliferative neoplasia. Vector transmission is suspected among dogs and wild canines, which are the primary reservoir hosts. This investigation was initiated to determine if pets and family members were infected with one or more *Bartonella *species.

**Methods:**

PCR and enrichment blood culture in *Bartonella *alpha Proteobacteria growth medium (BAPGM) was used to determine infection status. Antibody titers to *B. vinsonii *subsp. *berkhoffii *genotypes I-III and *B. henselae *were determined using a previously described indirect fluorescent antibody test. Two patients were tested sequentially for over a year to assess the response to antibiotic treatment.

**Results:**

Intravascular infection with *B. vinsonii *subsp. *berkhoffii *genotype II and *Bartonella henselae *(Houston 1 strain) were confirmed in a veterinarian and his daughter by enrichment blood culture, followed by PCR and DNA sequencing. Symptoms included progressive weight loss, muscle weakness, lack of coordination (the father) and headaches, muscle pain and insomnia (the daughter). *B. vinsonii *subsp. *berkhoffii *genotype II was also sequenced from a cerebrospinal fluid BAPGM enrichment culture and from a periodontal swab sample. After repeated courses of antibiotics, post-treatment blood cultures were negative, there was a decremental decrease in antibody titers to non-detectable levels and symptoms resolved in both patients.

**Conclusions:**

*B. vinsonii *subsp. *berkhoffii *and *B. henselae *are zoonotic pathogens that can be isolated from the blood of immunocompetent family members with arthralgias, fatigue and neurological symptoms. Therapeutic elimination of *Bartonella *spp. infections can be challenging, and follow-up testing is recommended. An increasing number of arthropod vectors, including biting flies, fleas, keds, lice, sandflies and ticks have been confirmed or are suspected as the primary mode of transmission of *Bartonella *species among animal populations and may also pose a risk to human beings.

## Background

When a genus of bacteria is discovered or, in the case of *Bartonella*, rediscovered; numerous clinical, microbiological and pathological concepts related to disease causation and microbial pathogenesis are sequentially redefined. Subsequently, the medical relevance of the genus undergoes continued maturation; as knowledge of the organism, the host immune response, diagnostic test sensitivity and specificity, treatment efficacy and epidemiology expand. Since the early 1990s, this paradigm of discovery and ongoing biological and medical redefinition has clearly been applicable to the genus *Bartonella*. Prior to 1990, only two pathogenic *Bartonella *species, *B. bacilliformis *and *B. quintana*, were known to exist. Since 1990, greater than 22 *Bartonella *species have been described, of which at least half have been implicated or confirmed as human pathogens [1,2].

*Bartonella vinsonii *subsp. *berkhoffii *was initially isolated from a dog with endocarditis in 1993 [3]. Subsequently, four genotypes of *B. vinsonii *subsp. *berkhoffii *were described, based upon analysis of blood samples from coyotes, dogs and foxes [4]. To date, genotype II has been the most frequently isolated genotype sequenced from dog and human blood samples [5-8]. Previously, *B. vinsonii *subsp. *berkhoffii *genotype II was documented in a healthy dog on 8 of 10 culture attempts spanning a 16-month period, thereby supporting the potential for persistent intravascular infection in pet dogs [9]. Although tick transmission of *B. vinsonii *subsp. *berkhoffii *has been suggested, the mode(s) of transmission among canines has not been determined [10]. In contrast to the canine reservoir for *B. vinsonii *subsp. *berkhoffii*, domestic and wild felids represent the primary reservoir in nature for *Bartonella henselae*, an organism transmitted among cats by fleas (*Ctenocephalides felis*); a factor that contributes to a worldwide distribution for this *Bartonella *sp. [1,2]. Similar to dogs, outwardly healthy cats can remain bacteremic with *B. henselae *for months to years [11,12]. However, despite what seems to be exceptional evolutionary adaptation of *B. vinsonii *subsp. *berkhoffii *in canines and *B. henselae *in felines, both of these two bacterial species can be pathogenic in both cats and dogs.

In this study, intravascular infection with *B. vinsonii *subsp. *berkhoffii *genotype II and *B. henselae *were foundin a veterinarian and his daughter. The father presented with progressive weight loss, muscle weakness and lack of coordination; his daughter had developed headaches, muscle pain and insomnia. Both individuals were being evaluated by a neurologist at the time of initial testing for evidence of *Bartonella *infection. Multiple courses of antibiotics were administered before the patients' clinical status improved and before microbiological, molecular and serological evidence of infection diminished or was negative.

## Patients, pets and methods

In October, 2007, the primary author was contacted by the father of a family residing in North Carolina, who requested Bartonella testing as a component of an IRB approved study (North Carolina State University Institutional Review Board, IRB#s 4925-03 and 164-08-05). For all family members and pets (Institutional Animal Care and Use Protocol 07-014-0) tested in this study, a previously described approach that combines PCR detection of *Bartonella *spp. DNA and enrichment culture of blood and serum samples in *Bartonella *alpha Proteobacteria growth medium (BAPGM) was used [8]. The three part BAPGM diagnostic platform incorporates PCR amplification of *Bartonella *spp. following direct DNA extraction from patient blood and serum samples, PCR amplification following enrichment culture in BAPGM for 7 to 14 days, and PCR from isolates obtained following BAPGM subculture inoculation onto trypticase soy agar with 10% rabbit blood. Agar plates are incubated for 4 weeks and checked weekly for evidence of bacterial growth. To assess for potential laboratory contamination, an un-inoculated BAPGM culture flask was processed simultaneously and in an identical manner with each batch of patient blood and serum samples tested. Specifically, while establishing cultures using a batch of samples in the biosafety hood, the top was removed from the BAPGM un-inoculated control flask until all patient samples had been processed. Methods used for testing sample cultures, including DNA extraction, PCR amplification targeting the *Bartonella *16S-23S intergenic spacer region (ITS), and sequencing procedures were performed using previously described methods [5-8]. Following the standard operating procedures in the Intracellular Pathogens Research Laboratory, sample preparation including BAPGM cultures and agar plate sub-inoculation, DNA extraction, PCR preparation and PCR amplification and analysis were performed in separate laboratory rooms to avoid culture as well as DNA contamination. In addition, negative and positive *Bartonella *DNA test control samples, consisting of bacteria-free blood DNA and DNA spiked with *B. henselae *genomic DNA at 0.5 genome copies per microliter, respectively, were used for each batch of DNA tested. For all results reported in this study, PCR products consistent in size with a *Bartonella *spp. (400-600 bp amplicon size) were sequenced to confirm the species and ITS strain. Sequences were aligned and compared with GenBank sequences using AlignX software (Vector NTI Suite 6.0, InforMax, Inc.).

Serology was performed using modifications of a previously described indirect fluorescent antibody test [13]. *Bartonella vinsonii *subsp. *berkhoffii *and *B. henselae *antibodies were determined following traditional immunofluorescence antibody assay (IFA) practices with fluorescein conjugated goat anti-human IgG. *Bartonella vinsonii *subsp. *berkhoffii *genotypes I, II and III and *B. henselae *(Houston I strain) were passed from agar grown cultures of each organism into DH82 (a continuous canine histiocytic cell line) cultures to obtain antigens that would seemingly be expressed by an intracellular bacteria localized to erythrocytes or endothelial cells within the vasculature. Heavily infected cell cultures were spotted onto 30-well Teflon coated slides (Cel-Line/Thermo Scientific), air dried, acetone fixed and stored frozen. Serum samples were diluted in phosphate buffered saline (PBS) solution containing normal goat serum, Tween-20 and powdered nonfat dry milk to block non-specific antigen binding sites. Patient sera were screened at dilutions of 1:16 to 1:64. All sera that remained reactive at a titer of 1:64 were further tested with twofold dilutions out to a final dilution of 1:8192.

## Results

### Father

The father was a 50-year-old veterinarian whose symptoms began in 2006 with arthralgias and fatigue, which became progressively severe over ensuing 18 months. He described pain and stiffness of the joints, muscles, and neck that were most severe in the morning but improved throughout the day. He did not have fevers, but he suffered from profound fatigue. He had also experienced an 80-pound weight loss, though this was partially intentional. Beginning in September 2007, and of greatest concern to the patient, was progressive difficulty maintaining his balance while standing or ambulating. His history was notable for extensive occupational and domestic animal exposure. International travel was minimal. He was initially evaluated by a neurologist, and because of his exposure to zoonotic pathogens he was referred for infectious disease evaluation. He had not received any empiric courses of antibiotics.

**Figure 1:**
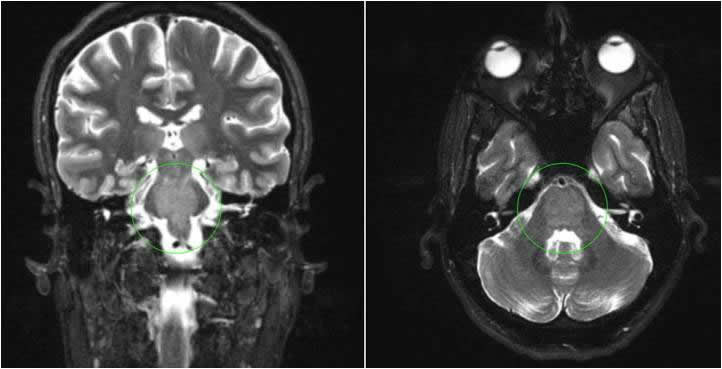
**Cranial T2-weighted MRI showing mildly increased signal throughout the pons (circled), as well as in the upper medulla to the left of midline**. Also noted was mild diffuse atrophy and a few nonspecific foci of increased T2 signal seen in the frontal lobes.

On physical examination, the patient had a blood pressure of 141/88 mm Hg, a pulse of 98 beats/min, and a temperature of 37°C. He was in no acute distress and had a normal sensorium. Notable abnormal findings included a positive Romberg sign and difficulty with heel-toe walking. Cranial nerves, muscle strength, sensation, and deep tendon reflexes were normal and symmetrical, and his funduscopic examination was normal. There was no lymphadenopathy, no organomegaly, and no rash. Lumbar puncture revealed normal cerebrospinal fluid indices and opening pressure. Magnetic resonance imaging of the brain was notable for an increase in signal intensity throughout the pons and upper medulla lateralizing to the left of the midline (Figure 1).

*Bartonella vinsonii *subsp. *berkhoffii *was amplified and sequenced directly from the initial EDTA-anti-coagulated blood sample obtained from the father; however, enrichment blood culture was PCR negative following a 7-day incubation period and a subculture (agar plate maintained for 4 weeks) failed to result in bacterial growth (Table 1). By IFA testing, the father was seroreactive to *B. vinsonii *subsp. *berkhoffii *genotypes II and III and *B. henselae *antigens. Based upon these findings, blood and cerebrospinal fluid were obtained for culture in BAPGM approximately 3 weeks later, at which time *B. vinsonii *subsp. *berkhoffii *was amplified and sequenced from both 14 day blood and cerebrospinal fluid enrichment cultures (7-day enrichment cultures were again PCR negative). Seroreactivity to *B. vinsonii *subsp. *berkhoffii *antigens remained essentially unchanged (within one dilution of previous results); however, there was a marked increase in the *B. henselae *antibody titer. The father reported a history of periodontal disease, which coincided with the onset of his illness. Sterile cotton swabs were used to obtain saliva and periodontal surface samples, after which ITS-PCR generated amplification products from both samples. Efforts to sequence the amplicon from saliva was not successful; however, *B. vinsonii *subsp. *berkhoffii *was amplified and sequenced from the periodontal swab. As *B. vinsonii *subsp. *berkhoffii *DNA was identified in three different sample sources (blood, CSF and periodontal surface) and at three different time points in the laboratory, he was treated for bartonellosis with doxycycline plus rifampin. During the first week of therapy he reported a worsening of symptoms, followed by gradual improvement. Following 3 weeks of antibiotics his *B. henselae *antibody had titer decreased fourfold, his *B. vinsonii *subsp. *berkhoffii *titers remained unchanged, and *B. henselae *(ITS Houston I strain) was amplified and sequenced from two 14-day BAPGM enrichment cultures (both EDTA and ACD-anti-coagulated blood samples were independently processed). Based upon this result, he received an additional 6 weeks of doxycycline plus rifampin. During the subsequent 11 months, PCR evidence of *Bartonella *infection was not detected in two additional blood cultures, the patient became non-seroreactive *B. henselae *and *B. vinsonii *subsp. *berkhoffii*. Post-treatment, the patient gradually regained body weight and no longer experienced arthralgias or neurological symptoms.

**Table 1:** Serological, culture and molecular test results for a 50-year-old veterinarian (the father) with chronic weight loss and progressive neurological dysfunction

	*Bartonella *IFA Reciprocal Titers			PCR/DNA Sequencing Results		
**Date/Sample (Father)**	***B. henselae***	***Bvb *Genotype II**	***Bvb *Genotype III**	**Direct Extraction**	**BAPGM Enrichment Culture**	**Subculture Isolate**

10-18-07 Blood	512	32	256	*Bvb *TII	Neg	NIO
11-02-07 Blood	8192	64	128	Neg	*Bvb *TII	NIO
11-05-07 CSF	NT	NT	NT	Neg	*Bvb *TII	NIO
12-11-07 Oral Swab	NT	NT	NT	Bvb TII	N/A	N/A
1-18-08 Blood	1024	32	128	Neg	*Bh *H1	NIO
5-27-08 Blood	<16	16	16	Neg	Neg	NIO
11-04-08 Blood	<16	<16	<16	Neg	Neg	NIO

Neg = DNA was not amplified using *Bartonella *16S-23S intergenic spacer (ITS) primers.

NIO = No isolate obtained by subculture following BAPGM (*Bartonella *alpha Proteobacteria growth medium) enrichment culture.

N/A = Not applicable

NT = Not tested

*Bvb*II = *Bartonella vinsonii *subsp. *berkhoffii *Genotype II by DNA sequencing

*Bh *H1 = *Bartonella henselae *ITS Houston I like strain by DNA sequencing

### Daughter

The 7 1/2 year old daughter of the above patient first sought medical care for a similar constellation of symptoms in October, 2007. Her illness began suddenly one morning with severe neck pain. Over the next month the pain gradually improved but never fully remitted, and she additionally developed headaches, low-grade fevers, and general malaise. Her symptoms evolved to include intermittent weakness of her legs and paresthesias, which were so debilitating that she was no longer able to attend school. She was seen by a pediatric neurologist, and her vital signs and physical exam were noted to be normal. She did not have any objective neurologic deficits.

**Figure 2:**
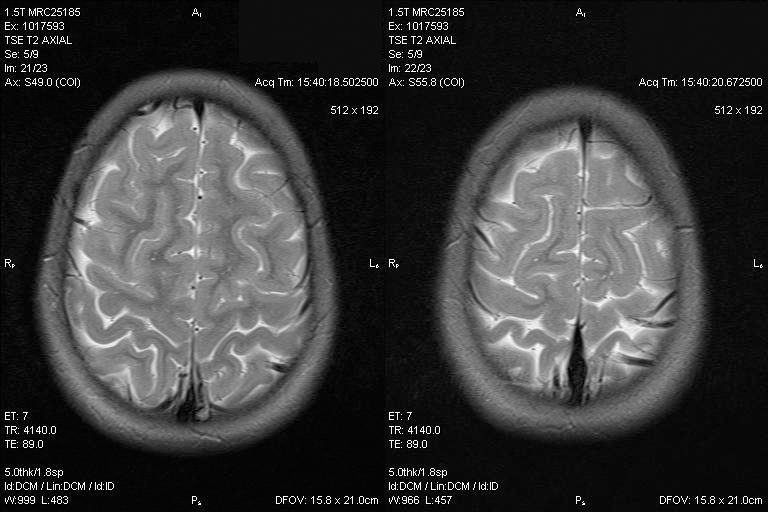
**Axial T2-weighted MRI of the daughter showing multiple, very small calcifications throughout the cerebrum in both white and gray matter, sparing the cerebellum**. These lesions were felt to be most consistent with a prior, inactive granulomatous process.

Based upon prior detection of *B. vinsonii *subsp. *berkhoffii *in the father's blood sample, a decision was made to test the daughter. The daughter was seroreactive to antigens of *B. vinsonii *subsp. *berkhoffii *genotypes II and III and to *B. henselae *(Table 2). In addition, *B. vinsonii *subsp. *berkhoffii *was amplified and sequenced from two BAPGM blood cultures obtained approximately three weeks apart. She was treated with a 6-week course of azithromycin, after which there was a fourfold decrease in the *B. henselae *antibody titer, though her *B. vinsonii *subsp. *berkhoffii *antibody titers remained unchanged. Despite initial symptomatic improvement, her symptoms recrudesced towards the end of this antibiotic course. *B. vinsonii *subsp. *berkhoffii *was again amplified and sequenced from a 14-day BAPGM enrichment culture and from the agar plate subculture (both the 7-day enrichment culture and subculture were PCR negative). She began a 9-week course of doxycycline, and at week 6, *Bartonella *spp. DNA was no longer detectable from the extracted blood sample, from the BAPGM enrichment blood culture, or from the agar plate. Due to continued intermittent neck and back pain the BAPGM diagnostic platform was repeated approximately 6 weeks later and a bacterial subculture isolate was obtained. Because no product was amplified using the ITS primers, different primers were used. An *Ochrobactrum *sp. was amplified and sequenced from a subculture isolate, using primers targeting the RpoB gene. She remained symptomatic while off therapy for the next two months, and though her symptoms were milder than before, they persisted and she was retreated with a 6-week course of doxycycline. During the subsequent two months antibody titers remained unchanged and two BAPGM blood cultures failed to result in PCR detection of *Bartonella *spp. infection. However, despite becoming seronegative to all test antigens, *B. henselae *(Houston 1 strain) was amplified and sequenced from a BAPGM enrichment culture obtained 3 months later. She had a relapse of neck pain several months later, but she did not receive antibiotics that time. One year after testing was initiated, the girl was no longer symptomatic and remained seronegative and blood culture negative. She suffered a minor, unrelated head injury during this time. A CT scan and subsequent MRI of the brain incidentally revealed multiple curvilinear calcifications in the left posterior parietal lobe along the periphery, throughout the cerebrum in both the gray and white matter, and sparing the cerebellum (Figure 2). These were thought to represent calcifications, possibly consistent with a prior granulomatous process. She was evaluated for infections known to induce granulomas, (toxoplasmosis, tuberculosis, histoplasmosis) but this workup was negative. The relationship of this radiographic finding to her *Bartonella *spp. infection is unclear. She remained off therapy, and has had no subsequent recurrence in symptoms during a 12-month follow-up period.

**Table 2:** Serological, culture and molecular test results for a 7 1/2 -year-old girl (the daughter) with progressive neurological dysfunction

	*Bartonella *IFA Reciprocal Titers			PCR/DNA Sequencing Results		
**Date/Sample** **(Daughter)**	***B. henselae***	***Bvb *Genotype II**	***Bvb *Genotype III**	**Direct Extraction**	**BAPGM Enrichment Culture**	**Subculture Isolate**

11-07-07 Blood	256	<16	128	Neg	*Bvb *TII	NIO
11-28-07 Blood	256	<16	64	Neg	*Bvb *TII	NIO
1-11-08 Blood	64	32	32	Neg	*Bvb *TII	*Bvb *TII
3-20-08 Blood	64	32	32	Neg	Neg	NIO
5-08-08 Blood	64	16	16	Neg	Neg	*Ochrobactrum *sp.
6-24-08 Blood	64	32	16	Neg	Neg	NIO
7-31-08 Blood	16	32	64	Neg	Neg	NIO
10-13-08 Blood	<16	<16	<16	Neg	*Bh *H1	NIO
12-01-2008 Blood	<16	<16	<16	Neg	Neg	NIO

Neg = DNA was not amplified using *Bartonella *16S-23S intergenic spacer (ITS) primers.

NIO = No isolate obtained by subculture following BAPGM (*Bartonella *alpha Proteobacteria growth medium) enrichment culture. *Bvb*II = *Bartonella vinsonii *subsp. *berkhoffii *Genotype II by DNA sequencing

*Bh *H1 = *Bartonella henselae *ITS Houston I like strain by DNA sequencing

#### Other Family Members

Within the family, the mother and two sons were reportedly healthy. As described for the father and daughter, *B. henselae *and *B. vinsonii *subsp. *berkhoffii *serology and BAPGM blood cultures were performed for the mother (March, 2008) and for the two sons (January, 2008). In these three individuals, antibody titers ranged from 1:32 to 1:128 to antigens of *B. henselae *and *B. vinsonii *subsp. *berkhoffii *genotypes II and III. *Bartonella *spp. was not isolated nor PCR amplified from blood or serum samples obtained from these three other family members.

#### Family Pets

The family had four domestic shorthair cats and two recently acquired one-year-old English Springer Spaniel littermates. Two other dogs, previously owned by the family, had been killed in an automobile accident, five months prior to the onset of illness in the father and daughter. All four cats had antibodies to *B. henselae *and *B. vinsonii *subsp. *berkhoffii *antigens by IFA testing, whereas antibodies were not detectable in serum samples from the two newly acquired dogs (Table 3). *Bartonella henselae *(ITS Houston 1 strain) was isolated from three of four cats. By DNA sequencing, the *B. henselae *ITS strains obtained from the father and daughter were identical to each of the *B. henselae *ITS sequences obtained from the three cats following direct extraction from blood, following BAPGM enrichment culture, and from agar plate derived isolates. The two English Springer Spaniel littermates were PCR negative for *Bartonella *spp. DNA following blood extraction, BAPGM enrichment cultures and no bacteria were isolated.

**Table 3:** Serological, culture and molecular test results for the household pets

	*Bartonella *IFA Reciprocal Titers			PCR/DNA Sequencing Results		
**Pet Designation**	***B. henselae***	***Bvb *Genotype II**	***Bvb *Genotype III**	**Direct Extraction**	**BAPGM Enrichment Culture**	**Subculture Isolate**

JE Cat	512	128	64	*Bh *H1	*Bh *H1	*Bh *H1
CO Cat	<16	128	<16	*Bh *H1	*Bh *H1	*Bh *H1
SN Cat	256	128	64	Neg	Neg	NIO
JA Cat	2048	2048	2048	*Bh *H1	*Bh *H1	*Bh *H1
TO Dog	<16	<16	<16	Neg	Neg	NIO
EM Dog	<16	<16	<16	Neg	Neg	NIO

Neg = DNA was not amplified using *Bartonella *16S-23S intergenic spacer (ITS) primers.

NIO = No isolate obtained by subculture following BAPGM (*Bartonella *alpha Proteobacteria growth medium) pre-enrichment culture.

*Bh *H1 = *Bartonella henselae *ITS Houston I like strain by DNA sequencing

## Discussion

In this study, we report the simultaneous detection of *B. vinsonii *subsp. *berkhoffii *infection in two family members who were experiencing neurological dysfunction. *Bartonella vinsonii *subsp. *berkhoffii *is an important emerging intravascular pathogen that has been isolated from patients with endocarditis, arthritis, neurological disease and vasoproliferative neoplasia [5,6,14,15]. In the current case report, both the father and daughter were infected with *B. vinsonii *subsp. *berkhoffii *genotype II strains and both were either co-infected or sequentially infected with *B. henselae*. Considering the sequential serological test results obtained for the father, it seems likely that he was co-infected, when the first blood culture was obtained. Initial *B. henselae *antibody titers for the father were 8 to 12 fold higher than the titers obtained using *B. vinsonii *subsp. *berkhoffii *genotype II antigens, whereas only a 6 fold difference was found in the initial two serum samples from the daughter. As there is a less convincing serological association supporting co-infection in the daughter and as three of four cats in the household were *B. henselae *bacteremic during the period in which the father and daughter were being treated for their *B. vinsonii *subsp. *berkhoffii *infections, it is possible that sequential infection with two different *Bartonella *spp. was documented in the daughter during the course of this study. As the isolation and molecular detection of these bacteria from patient samples remains microbiologically challenging, we were unable to clearly establish whether either patient was co-infected or sequentially infected at various time points.

Previously, we described the preferential amplification of one *Bartonella *sp. when two or more species are present in the extracted sample [16]. The mechanism(s) responsible for preferential amplification of one bacteria when DNA of two species is present in a patient sample is unclear, but mechanisms could include the relative concentrations of DNA of the respective organisms in the sample at the time of DNA extraction for PCR amplification, or selective amplification of one DNA sequence over the other one, when comparable template concentrations are present. Targeting multiple *Bartonella *genes can enhance the possibility of detecting co-infection in patient samples [17-19]. Potentially, PCR primers targeting different gene fragments preferentially amplify different *Bartonella *spp. in co-infected individuals. It is also possible that BAPGM enrichment culture preferentially selects for the growth of one *Bartonella *spp. in a co-infected individual. Therefore both individuals may have been co-infected with *B. vinsonii *subsp. *berkhoffii *and *B. henselae *at the outset of this study.

As the sequences of the *B. henselae *strains obtained from the father and daughter's blood cultures were identical (*B. henselae *ITS strain Houston1) to the blood culture strains obtained from the three cats, the family cats were the presumed source of this infection.

Although the mother and two sons were healthy and blood culture negative when tested on a single occasion, all three had serological evidence supporting prior exposure to *B. henselae*. The two newly acquired young dogs were not seroreactive to *Bartonella *antigens and both were blood culture negative; therefore it seems unlikely that these dogs played a role in transmission of *B. henselae *or *B. vinsonii *subsp. *berkhoffii *in the family. Although cats are the primary reservoir host, *B. henselae *has been isolated by blood culture from dogs and sequenced from dog saliva [8,9]. It is possible that an arthropods or the two older dogs that had died prior to initiation of this study were the source of *B. vinsonii *subsp. *berkhoffii *infection, as historically this organism has been isolated from domestic and wild canines and humans [1,2]. Infection with *B. vinsonii *subsp. *berkhoffii *was recently described in a cat with recurrent osteomyelitis that was bacteremic over a 15-month time period [20]. Therefore, cats may be able to maintain a persistent *B. vinsonii *subsp. *berkhoffii *bacteremia and potentially serve as a source of bacterial transmission to humans [14]. Efforts to amplify *B. vinsonii *subsp. *berkhoffii *from the cats in this study using subspecies-specific primers were not successful. Over 100 years ago, *B. quintana *was transmitted to human volunteers, when saliva from a febrile patient was applied to escharified skin [21]. As saliva obtained from soldiers with trench fever apparently contains viable, infectious *Bartonella quintana*, oral transmission of *B. vinsonii *subsp. *berkhoffii *from the father or a pet cat to the daughter through close family contact cannot be ruled out. *Bartonella *spp. DNA has now been reported in the saliva of cats, dogs and humans [22,23]. Clearly, additional data is needed to define the risk factors for *Bartonella *spp. transmission to humans and to their pets, but it seems prudent to recommend hygienic measures after contacting pet and perhaps human saliva.

The father in this report is the second patient in which enrichment culture enhanced the molecular detection of a *Bartonella *spp. in cerebrospinal fluid. In a previous study, *B. henselae *was repeatedly detected by blood or cerebrospinal fluid culture in a 23 year-old girl who developed progressively severe seizures following a history of cat scratch disease [6]. In both patients, cerebrospinal fluid analyses were reported to be within normal limits; however, inadvertent contamination of the sample with blood cells cannot be ruled out. As *Bartonella *spp. appear to target vascular endothelial cells, it is possible that *B. vinsonii *subsp. *berkhoffii *and *B. henselae *contributed to the nonspecific area of vascular injury reported on the father's MRI.

The use of an optimized insect-based cell culture growth medium can facilitate the isolation or enhanced molecular detection of *Bartonella *spp. following culture-enrichment of patient samples prior to performing PCR [5,6,8,14,18]. The enhanced diagnostic utility of the enrichment approach is best illustrated in Table 2, where *B. vinsonii *subsp. *berkhoffii *and *B. henselae *DNA were never directly amplified from extracted blood samples obtained from the daughter and were only detectable by PCR following enrichment culture. In our experience, at least a 7 to 14-day incubation period is required before the enriched sample is obtained from liquid BAPGM for DNA extraction. At no time during this study was *Bartonella *spp. DNA amplified from a DNA extraction control, a BAPGM un-inoculated enrichment culture control or a subculture agar plate control. Although not detailed in the table, it is not unusual for 7-day enrichment cultures and subcultures to be PCR negative for *Bartonella *spp. DNA, whereas the respective organism can be amplified and sequenced after a 14-day incubation period from the enriched liquid culture, the agar plate isolate, or both. Due to the high level of *B. henselae *bacteremia generally found in cats, the same strain could be detected in each cat following direct extraction of the blood sample, enrichment culture and the agar plate isolates (Table 3). Unfortunately, despite the enhanced utility of enrichment culture for the molecular microbiological diagnosis of *Bartonella *infection, obtaining viable agar plate isolates after subculture from liquid BAPGM at 7 or 14 days post-incubation remains technically difficult. In this study, only one *B. vinsonii *subsp. *berkhoffii *isolate was obtained from the daughter's post-antibiotic blood culture after a 14-day enrichment period, whereas the 7-day BAPGM enrichment culture and subculture were both PCR negative. An isolate was never obtained from the father; however as described above, using the same BAPGM enrichment platform, *B. henselae *isolates were obtained from three of the four cats in the household. As overtly healthy cats can maintain a high-level of bacteria in systemic circulation for months to years, isolation of *B. henselae *form cat blood samples is comparatively easy to achieve, as compared to isolation using the same approaches from dog or human blood samples [11,12]. Failure to obtain stable *Bartonella *isolates is a major patient management limitation, as it prevents routine testing for antibiotic sensitivity and resistance of specific isolates at time points prior to and following antibiotic administration. This was of particular concern for these two patients, as *B. henselae *DNA was still sequenced from an enrichment culture of the father's blood following a 3-week course of doxycycline and rifampin and *B. vinsonii *subsp. *berkhoffii *was detected in the girl's blood following a 6-week course of azithromycin. Based upon negative post-antibiotic blood cultures and a decremental decrease in antibody titers to non-detectable levels, antibiotic treatment appeared to correlate with microbiological elimination of *B. vinsonii *subsp. *berkhoffii *and *B. henselae *infections, cessation of antibody production, and with the eventual clinical resolution of illness in both patients.

The father reported a recent history of severe periodontal disease. Two previous molecular microbiological studies identified *Bartonella *spp. DNA in subgingival samples from patients with periodontitis. [24,25]. In addition, other investigators have detected *B. henselae *DNA and *B*. *quintana *DNA in the parotid salivary glands of an immunocompetent woman and man, respectively and *B. quintana *in the dental pulp of a homeless man [26-28]. After which *B. vinsonii *subsp. *berkhoffii *genotype II was successfully sequenced from the periodontal swab, but attempts to sequence the amplicon obtained from the salivary swab were not successful (Table 1). As identical techniques were used, this result might be explained by a higher concentration of *B. vinsonii *subsp. *berkhoffii *DNA at the periodontal surface, as compared to dilution of bacterial DNA targets floating in saliva in the oral cavity. In our laboratory, a *B. henselae *SA2 strain (2.5 copies/reaction) is used as the source of positive control DNA for the ITS PCR reaction; therefore, contamination with positive control DNA could not explain any PCR results obtained in these two patients. As over 500 species of bacteria have been estimated to inhabit the oral cavity, BAPGM enrichment culture was not attempted because rapidly growing organisms would negate efforts to increase Bartonella numbers via the enrichment process [29,30]. It is also possible that detection of *B. vinsonii *subsp. *berkhoffii *in close approximation of the periodontal surface reflects passive leakage of bacteria through inflamed and compromised vascular tissues or alternatively the establishment of an active foci of infection that contributed to the recent history of periodontitis. Detection of DNA in the oral cavity does not confirm the presence of viable bacteria; however, caution should be exercised by dentists and physicians when examining the oral cavity of an individual with chronic *Bartonella *spp. bacteremia.

## Conclusions

*B. vinsonii *subsp. *berkhoffii *and *B. henselae *are zoonotic pathogens that can be isolated from the blood of immunocompetent family members with arthralgias, fatigue and neurological symptoms. Therapeutic elimination of *Bartonella *spp. infections can be challenging, and follow-up testing is recommended. An increasing number of arthropod vectors, including biting flies, fleas, keds, lice, sandflies and ticks have been confirmed or are suspected as the primary mode of transmission of *Bartonella *species among animal populations and may also pose a risk to human beings.

## Competing interests

In conjunction with Dr. Sushama Sontakke and North Carolina State University, Dr. Breitschwerdt holds U.S. Patent No. 7,115,385; Media and Methods for cultivation of microorganisms, which was issued October 3, 2006. He is the chief scientific officer for Galaxy Diagnostics, a newly formed company that provides diagnostic testing for the detection of *Bartonella *species infection in animals and in human patient samples. Dr. Ricardo Maggi performed all molecular microbiological testing reported in this study and is the Scientific Technical Advisor and Laboratory Director for Galaxy Dx.

## Authors' contributions

EBB was involved in all aspects of this study, including generation of the initial draft of the manuscript; RGM performed all blood cultures, PCR, sequencing and molecular data analyses, PML and CWW were responsible for patient evaluation, medical record review and patient follow-up, BCH and JMB were responsible for serological testing. All authors contributed to the content and approved the final manuscript.
